# Intraprocedural Findings and Outcomes of Redo Procedures After Voltage-Guided Ablation of Persistent Atrial Fibrillation

**DOI:** 10.3390/jcm15083005

**Published:** 2026-04-15

**Authors:** Maxi Hartmann, Duc Nguyen, Violeta Mattea, Frank Steinborn, Mykhaylo Chapran, Ralph Surber, Kourosh Vathie, Mohamad Assani, Hussam Hamo, Hamzeh Alsous, Lisa C. Costello-Boerrigter, Jens Martin Kittner, Alexander Lauten, Christian Schulze, Anja Schade

**Affiliations:** 1 Department of Cardiology/Interventional Electrophysiology, Helios Hospital Erfurt, Nordhäuser Str. 74, 99089 Erfurt, Germany; vm77@gmx.net (V.M.); frank.steinborn@helios-gesundheit.de (F.S.); alexander.lauten@helios-gesundheit.de (A.L.); 2 Department of Cardiology, St. Georg Klinikum Eisenach, Mühlhäuser Str. 94, 99817 Eisenach, Germany; duc.nguyen@helios-gesundheit.de; 3 Department of Cardiology, Helios Frankenwaldklinik Kronach, Friesener Str. 41, 96317 Kronach, Germany; mykhaylo.chapran@helios-gesundheit.de; 4 Department of Cardiology, Internal Intensive Care and Angiology, University Clinic Jena, Am Klinikum 1, 07747 Jena, Germany; ralph.surber@med.uni-jena.de (R.S.); christian.schulze@med.uni-jena.de (C.S.); 5 Department of Cardiology, Helios Klinikum Meiningen, Bergstraße 3, 98617 Meiningen, Germany; kourosh.vathie@helios-gesundheit.de; 6 Department of Cardiology, Klinikum Bad Hersfeld, Seilerweg 29, 36251 Bad Hersfeld, Germany; mohamad.assani@helios-gesundheit.de; 7 Department of Cardiology, Helios Klinikum Bonn/Rhein-Sieg, Von-Hompesch-Stra. 1, 53123 Bonn, Germany; hussam.hamo@helios-gesundheit.de; 8 Department of Cardiology, Diakonie Klinikum Jung-Stilling, Wichernstr. 40, 57047 Siegen, Germany; hamzeh.alsous@helios-gesundheit.de; 9 Department of Cardiology, Heart Center, Zentralklinik Bad Berka, Robert-Koch-Allee 9, 99438 Bad Berka, Germany; elizabeth.costello.extern@zentralklinik.de; 10 Department of Gastroenterology, Hepatology, Endocrinology, Diabetology and Rheumatology, Helios Hospital Erfurt, Nordhäuser Str. 74, 99089 Erfurt, Germany; jens.kittner@helios-gesundheit.de; 11 Department of Cardiology II, Rhythmology and Interventional Electrophysiology, RÖHN Clinic “Campus Bad Neustadt”, Von-Gutteberg-Straße 11, 97616 Bad Neustadt a. d. Saale, Germany; anja.schade@campus-nes.de

**Keywords:** atrial fibrillation redo ablation, voltage-guided ablation, reconnection rate, low voltage zones, pulmonary vein isolation, persistent atrial fibrillation ablation, atrial fibrosis

## Abstract

**Background/Objectives:** Pulmonary vein isolation (PVI) is the gold standard for atrial fibrillation (AF) ablation. Recently, a randomized study showed that adding voltage-guided ablation (VGA) in persistent AF cases was beneficial. The aim of the present study was to evaluate the safety, efficacy and predictors of success of redo procedures after VGA in an exclusively persistent AF cohort. **Methods:** Data are derived from the prospective Erfurt AF ablation registry. Starting in 2015, ablation procedures were performed using CARTO3D and VGA. Patients receiving their first redo procedure between 01/2015 and 08/2022 were included. Follow-up included 72 h Holter ECG or device interrogation, ECG, symptom-triggered event recording, and questioning at 3 and 12 months after the redo procedure. The primary endpoint was freedom of recurrence of AF or atrial tachycardia (AT) without drugs between 3 and 12 months. **Results:** Altogether, 683 patients received a first VGA between January 2015 and May 2022, and 77 patients had their first redo procedure occurring 20 ± 17 months after the first procedure. During the first redo procedure, reconnected PVs were found in 44%, reconnected lines in 23% and new or progressive LVZs in 57% of patients. Complications occurred in two patients (2.6%). During follow-up one patient died, and one did not participate due to aphasia. It was found that 69% were free of recurrence and 61% were free of recurrence off drugs. Patients with recurrence were older than those without recurrence off drugs (73 ± 6 versus 69 ± 9 years, *p* = 0.049). **Conclusions:** Redo procedures after VGA in persistent AF have comparatively good results; although, many patients have progressive fibrosis.

## 1. Introduction

Pulmonary vein isolation (PVI) is the gold standard of atrial fibrillation (AF) ablation [1].

In persistent AF, success rates after a PVI-only approach are modest [2]. This might be due to the greater extent of left atrial (LA) fibrosis observed in these cases.

In 2014 Rolf et al. reported beneficial effects from an ablation strategy that additionally ablated LA low-voltage zones (LVZs), which represent fibrotic areas [3]. Recently, a randomized study found increased success rates with the addition of voltage-guided ablation (VGA) versus PVI alone in patients with persistent AF [4].

However, other randomized studies failed to find improved success in mixed or paroxysmal AF cohorts using different VGA strategies [5,6].

The higher the number of lines, the greater the risk is of causing gaps and promoting regular atrial tachycardias (ATs), but the use of the ablation index (AI) in combination with the “CLOSE” concept produces more contiguous lesions and so increases the success rate and durability of PVI [7]. Using a population of persistent AF patients, we aimed to evaluate the complications and efficacy of redo procedures after VGA. Given that use of the “CLOSE” concept might also increase the durability of other lines, we additionally aimed to evaluate its influence on reconnection rates.

## 2. Methods

### 2.1. Study Cohort

All data came from the prospective Erfurt Atrial Fibrillation Ablation Registry, which included all AF ablation procedures. Starting in 2015, all ablation procedures for persistent AF were generally performed using CARTO3D and VGA. Consecutive patients receiving their first redo procedure after VGA for persistent AF between January 2015 and August 2022 were included in the present study.

High-power short-duration ablation (HPSD 50W) was used at Helios Hospital in Erfurt from 19 November 2021 until now.

### 2.2. First Ablation Procedure

The methodology of the first ablation procedure has previously been described in detail [8]. In brief, procedures took place under conscious sedation with propofol and sufentanil. After double transseptal access, a voltage map was created during sinus rhythm (SR) using CARTO 3 and a Lasso^®^ 2515 Nav catheter (Johnson & Johnson, New Brunswick, NJ, USA). Significant LVZs were defined by voltage points < 0.5 mV in an area measuring at least 5% of the LA endocardial surface. Thermocool Smarttouch^®^ SF^TM^ catheter (Johnson & Johnson, New Brunswick, NJ, USA) was used for circumferential PVI and VGA. Twenty minutes after isolation was achieved, bidirectional conduction block was proven.

VGA was performed as described earlier [8]. In brief, small LVZs were ablated completely, lines were applied to isolate larger LVZs, and strategic lines were drawn to avoid critical isthmi. Bidirectional block was carefully proven at all applied lines.

**Figure 1:**
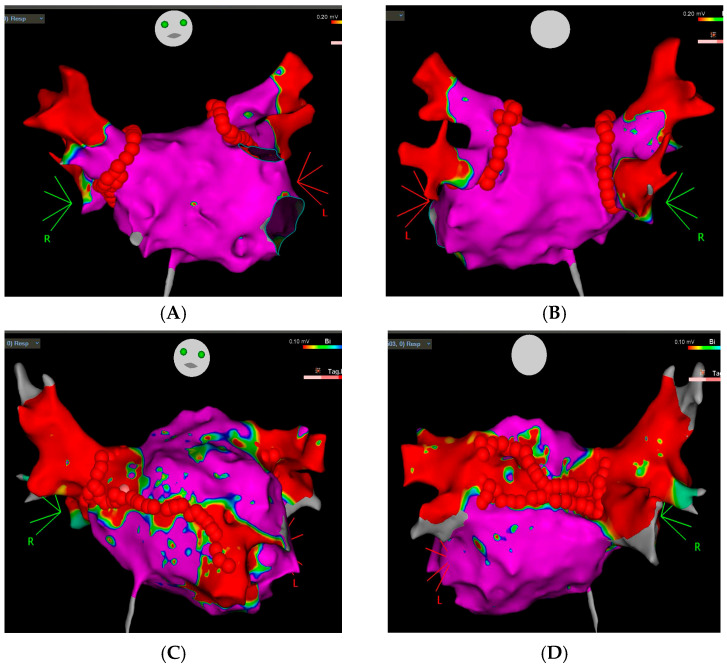
Examples of voltage-guided ablation strategies. (**A**) PVI without ablation of LVZs, anterior view; (**B**) PVI without ablation of LVZs, posterior view; (**C**) Anterior Mitral Line; (**D**) Modified Box Lesion.

Figure 1 shows VGA strategies (A PVI without ablation of LVZs, anterior view; B PVI without ablation of LVZs, posterior view; C Anterior Mitral Line; D Modified Box Lesion).

A power of 30–35 watts (flow rate 15 mL/min) was used for anterior wall RF ablation and 20–25 watts (flow rate 8 mL/min) for the posterior wall. Point-by-point ablations were performed targeting contact force of 5 to 40 g for 20 to 30 s or until local signals disappeared for contact force-guided (CFG) ablations. After introduction of AI, an ablation approach similar to the previously published “CLOSE” concept was used, such that AI targets of ≥500 anteriorly and of ≥350 or 400 posteriorly were used to set point by point lesions. The CARTO 3 VISITAG™ (Johnson & Johnson, New Brunswick, NJ, USA) module was used for the automated display of the RF applications. VISITAG™ parameters were tag size of 3 mm, location stability of 2.5, minimum time of 3 s, force over time of 25%, minimum force of 3 g. Gaps between the visitags were avoided, so that an interlesion distance of ≤6 mm was reached like described by Duytschaever et al. [7].

### 2.3. First and Second Redo Ablation Procedure

After exclusion of an LA thrombus using transesophageal echocardiography, the ablation procedures were performed under conscious sedation with propofol and sufentanil.

After a fluoroscopy- and pressure-guided transseptal puncture, two sheathes were inserted into the LA via the same puncture site. LA anatomical and voltage maps with depiction of all PVs were created during SR using CARTO 3 and a Lasso^®^ 2515 Nav Eco (Johnson & Johnson, New Brunswick, NJ, USA) or a Pentarray^®^ Nav high-density mapping catheter (Johnson & Johnson, New Brunswick, NJ, USA) in combination with a steerable long sheath (Agilis, Abott, Plymouth, MN, USA).

Bidirectional block of PVs was confirmed including additional adenosine testing after 20 min waiting time. In case of PV reconnection, PV re-isolation was performed as the first step. Thereafter, bidirectional conduction block of LA lines was verified. In the case of reconnected lines, the earliest site of activation behind the line was mapped and the ablation started there. In case of new or progressive LVZs, VGA followed the same principles as described for the initial procedures [8]. In the case of an ongoing AT at the time of procedure start, the AT was mapped and entrainment pacing was used to determine the mechanism. AT was ablated before the SR map of voltage and controls of PVs and lines were performed.

In cases where AF was present at the beginning of the procedure, electro-cardioversion was performed before mapping. If AF recurred immediately, then the first map was performed in AF. After repeat PVI, a second cardioversion attempt was made, and the final voltage map was performed in SR before further ablation was done.

Finally, after waiting time, all new lines were examined again for bidirectional conduction block using differential pacing. Ablation energy settings and lesion visualization followed using the same methods as in the first procedures. In some of the patients, high power short duration ablation (HPSD) with 50 W was used with the same AI targets, as described above.

After removing all sheathes, a Z-suture and a compression bandage were applied.

### 2.4. Follow-Up

Follow-up visits took place 3 and 12 months after each ablative procedure. Follow-up included a patient interview, ECG, and 72 h Holter ECG or cardiac electronic device interrogation. Additionally, symptom-triggered event recording was carried out.

The first 3 months were defined as a blanking period meaning that recurrences during this time space do not count for the evaluation. Patients were advised to stop specific antiarrhythmic drugs 2 months after the ablation. The primary endpoint was freedom of AF or AT lasting longer than 30 s off drugs.

### 2.5. Statistical Analysis

To analyze the collected data, we used Stata IC16.1. Metric parameters are listed by mean value ± standard deviation (M ± SD).

For categorial parameters, absolute and relative frequencies are depicted. Group comparisons of metric parameters were performed using Mann–Whitney-U test. In the case of categorical parameters, the Fisher exact test was used.

Statistically significant results were accepted with a two-sided *p* < 0.05.

Logistic regression was used to analyze the effect of age and one additional parameter on recurrence of AF or AT.

## 3. Results

### 3.1. Description of the Cohort

Altogether, 683 persistent AF cases received their first VGA for persistent AF during the inclusion period. After the initial procedure, 69% of patients (468/683) had complete follow-up. Of these patients 19% had recurrence by the end of the 12-month follow-up. The recurrence rate was not significantly different between patients with and without LVZs (15% vs. 12%; *p* = 0.48). A first redo procedure after VGA was performed in 77 patients (mean age 70 ± 8 years, male sex in 51% and LAESVI 43 ± 13 mL/m2, mean LVEF 54 ± 9%). The mean interval between the initial ablative procedure and first redo procedure was 20 ± 17 months.

Baseline data for the initial and first redo procedures are presented in Table 1. Baseline parameters of the first redo cohort did not differ significantly from the initial procedure cohort.

### 3.2. Intraprocedural Findings of the First Redo Procedure

In 43% of patients (33/77) atypical atrial flutter was diagnosed intraprocedurally or before the redo procedure.

PV reconnection was found in 44% of patients (34/77) and in 20% of PVs (60/297). Per vein analysis revealed reconnection in 26% of LSPV (18/69), 17% of LIPV (12/69), 20% of RSPV (15/76) and 20% of RIPV (15/76).

Reconnected lines were found in 23% of patients (18/77) and in 45% of previously applied lines (25/56). Altogether, reconnection affected 35% of roof lines (7/20), 29% of posterior lines (2/7), 54% of anterior mitral lines (12/22) and 57% of septal mitral lines (4/7). New or progressive LVZs were detected in 57% of patients (44/77).

In 16.9% of patients (13/77) we found new LVZs only, without reconnection of PV or lines.

“CLOSE”-like concept was used during the initial ablation procedure in 36.4% of patients (28/77). The rate of patients with PV reconnection at the timepoint of the first redo procedure was not significantly different with 39% (11/28) after “CLOSE”-like ablation versus 47% (23/49) after CFG (*p* = 0.298).

The rate of reconnected lines was also not significantly different following “CLOSE”-like ablation with 44% of applied lines (10/23) versus CFG with 46% of applied lines (15/33); *p* = 0.871.

### 3.3. Procedural Data of First Redo Procedure

“CLOSE”-like ablation was performed in 69% of patients (53/77). HPSD50W in turn was used in 21% of patients (16/77).

During the first redo procedure 27% of patients (21/77) received a Re-PVI alone, in 45% of patients (35/77) only LVZs were ablated, in 22% (17/77) both approaches were necessary and in 6% (5/77) other regions were ablated.

**Figure 2:**
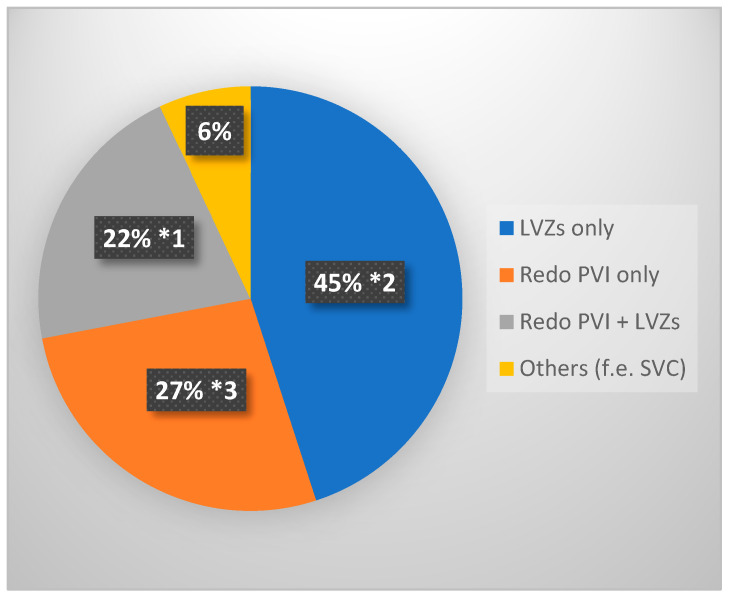
Ablation techniques in first redo procedures. *1 in one case additional ablation of CTI. *2 in two cases additional ablation of SVC; in one case: CTI. *3 in one case additional ablation of SVC. LVZs: low-voltage zones. PVI: pulmonary vein isolation. SVC: superior vena cava. CTI: cavo-tricuspid isthmus.

Figure 2 shows ablation techniques in first redo procedures.

Table 2 summarizes intraprocedural findings and ablation techniques used in both procedures.

The procedure time was on average 217 ± 68 min, the fluoroscopy time was 10 ± 5 min. The dose area product counted 7 ± 7 Gy/cm^2^. The procedure times were significantly shorter when “CLOSE”-like ablation was performed than in CF-guided ablation (158.7 ± 37.6 vs. 222.4 ± 67.8 min, *p* < 0.001).

The fluoroscopy times were significantly shorter with “CLOSE”-like ablation as well (9.1 ± 5.4 vs. 12.3 ± 4.3 min, *p* = 0.001).

### 3.4. Complications of the First Redo Procedure

Relevant complications occurred in two patients. In one patient a transient ischemic attack occurred, and another patient had sinus arrest requiring pacemaker implantation. Notably, there was no stroke, no pericardial tamponade, no atrioesophageal fistula, and there were no groin complications necessitating intervention.

### 3.5. Follow-Up After First Redo Procedure

During follow-up, one patient died. Another patient was not willing to take part in regular follow-up examinations because of an aphasia. Rhythm analysis was based on pacemaker interrogation in 12% of patients (9/75), 72 h Holter ECG in 65% (49/77) and ECG only in 19% of patients (14/75).

Looking at the 12-month success rate after the first redo, 52 out of 75 patients (69%) were free of recurrence and 46 out of 75 patients (61%) were free of recurrence off drugs.

### 3.6. Predictors of Recurrence After the First Redo Procedure

Patients with recurrence after the first redo procedure were significantly older than those without recurrence off drugs (73 ± 6 versus 69 ± 9 years, *p* = 0.049). The number of lines applied per patient during the initial procedure was higher in the recurrence group (1.0 vs. 0.5, *p* = 0.082). No other differences between the groups could be identified (Table 3). Multivariate analysis supported age as the only significant predictor of recurrence (*p* = 0.032), although the small number of patients limits the values of this finding.

The recurrence rate after the first redo procedure was not significantly different between procedures using “CLOSE”-like concept or CFG (16/46 (34.8%) vs. 7/23 (30.4%), *p* = 0.791).

Recurrence rate did not significantly differ between the group ablated with HPSD50W (50%) and the group ablated with lower power settings (29%) (*p* = 0.185).

### 3.7. Patients with Recurrences Within 12 Months After the First Redo Procedure

Out of the group of patients with recurrence within 12 months after the first redo procedure, 26% (6/23) underwent a second redo procedure.

**Figure 3:**
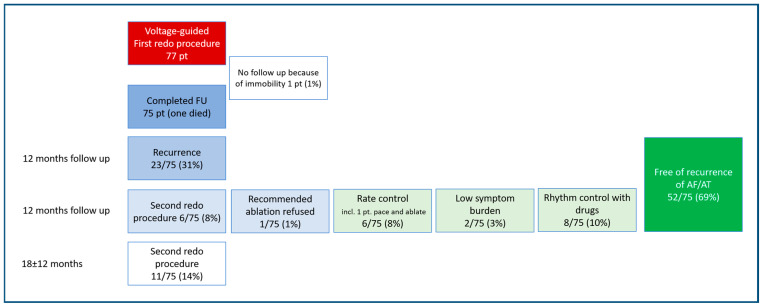
Flow chart first and second redo procedure. AF: atrial fibrillation. AT: atrial tachycardia. FU: follow-up. pt: patients.

Figure 3 shows the details of follow-up of the patient cohort who underwent a first redo procedure and the treatment actions in case of recurrence after the first redo procedure.

### 3.8. Procedural Data of Second Redo Procedures

Out of the 77 patients who underwent a first redo procedure, altogether 11 patients underwent a second redo procedure. In six of these 11 patients, the second redo took place within 12 months after the first redo procedure, and in the remaining five patients the second redo took place one year or more after the first redo procedure. Altogether, the mean time between the first redo and the second redo procedure was 18 ± 13 months.

The baseline parameters of the second redo procedure are listed in Table 4.

In 18% of patients (2/11), we found reconnected PVs; in 64% of patients (7/11), reconnected lines could be identified. Progressive or new LVZs occurred in 54% of patients (6/11). Relevant complications occurred in only one patient, specifically, AV block III° requiring a temporary pacemaker lead but which resolved spontaneously.

Detailed intraprocedural findings and measures are listed in Table 5.

### 3.9. Follow-Up of Second Redo Procedures

Follow-up in connection to the second redo procedures took place after 11 ± 4 months. Rhythm monitoring included 72 h Holter ECG monitoring in 54% of patients (6/11), 24 h Holter ECG monitoring in 9% (1/11), and 12 lead ECG in 27% (3/11) of patients. One patient died before the 12-month follow-up.

The results showed a freedom of recurrence of 70% (7/10). The freedom of recurrence off drugs in turn was 50% (5/10).

One of the patients out of the recurrence group received a third redo procedure one year after the second redo procedure. The intraprocedural results showed two reconnected lines and progressive substrate. One year after the fourth ablation in total, the patient was free of recurrence off drugs.

## 4. Discussion

### 4.1. Main Findings

This is the first study analyzing in detail intraprocedural findings and success rates of first and second redo ablations after VGA in a purely persistent AF cohort.

Of the initial persistent AF patient cohort (*n* = 683), 11% underwent a first VGA redo procedure.

Reconnected PVs were found in 44% of patients and reconnected lines in 23% of patients. New or progressive LVZs were detected in 57% of patients.

After the first redo procedure, 69% of patients were free of recurrence and 61% were free of recurrence off drugs. The only significant predictor of recurrence was age. A second redo procedure showed long-term freedom of recurrence in 70% and in 50% off drugs.

### 4.2. Intraprocedural Findings

#### 4.2.1. Reconnection of PVs and Lines

Expected mechanisms of recurrences after VGA are PV reconnection, reconnection of lines, and progressive LVZs as a correlate for fibrosis.

In our study the reconnection rates of PVs were comparatively low using CFG ablation or AI-guided “CLOSE”-like procedures. Consistent adherence to waiting time and a close set of points using CFG ablation might have contributed to the good results.

Reconnected lines were found in 23% of patients and in 45% of applied lines. The only study which examined a relevant number of redo procedures after VGA is the one of Huo et al. but included a significant rate of paroxysmal cases [4]. They found line reconnection in only 7% of patients. Their lower rate might be explained by a lower rate of drawn lines in the initial procedure as paroxysmal AF cases have lower rates of LVZs. The rate of reconnected lines based on applied lines in the initial procedures is not reported in their study.

Similar to our study, a meta-analysis including 1643 patients found that in 63% of cases there was reconnection in redo procedures after posterior wall isolation [9].

As no systematic re-mapping of the initial VGA cohort was performed, we do not know the rate of reconnected lines in our initial ablation cohort. Whereas, in earlier studies, durability of PVI lesions increased with CFG and “CLOSE” concept, the reconnection rates of LA lines remain unsatisfying.

Therefore, developing techniques to apply durable ablation lines in the LA is an important challenge to be addressed. New catheter designs and pulsed field ablation devices are under development to allow safer and more effective lesions.

#### 4.2.2. Low-Voltage Zones

In addition to reconnection as a potential mechanism for AF recurrence, we also found a high rate of LVZ progression and new LVZ development. The increase in LVZs reflects disease progression in our older cohort, with a mean age of 70 ± 8 and dilated LA. The rate of new LVZs (35%) was comparable to the rate observed by Huo et al. in a mixed cohort of paroxysmal and persistent AF cases [4]. Altogether, we observed new LVZs or LVZ progression in 57% of patients. This result is in line with results of Marcon et al., who found a decrease in voltage in non-ablated LA-regions in 61% of a mixed AF cohort after PVI [10]. Obviously, low voltage progression takes place independent of the initial ablation strategy and is a relevant cause for recurrences. However, an important observation of our study is, that in about 30% of our exclusively persistent AF redo cohort, PVI remained the only ablation strategy for initial ablation and first redo procedure.

### 4.3. Complications of Redo Procedure

One of the most important observations of our study is that complex redo procedures after VGA can be performed with a very low complication rate of 2.6%, which is comparable to published data regarding initial ablations [11]. Although we included patients who were 10 years older on average, our complication rate was comparably low to other cohorts with limited ablation concepts [12].

### 4.4. Success Rates

Data regarding redo ablation in persistent AF cases is rare and only observational data have been published [13]. We found encouraging success rates for redo procedures after VGA, with a 12-month freedom of recurrence of 69% (and 61% off drugs). This success rate was comparable to other studies in patients with persistent AF, which found freedom of recurrence of AF/AT to range from 58 to 66% [4,12,14]. Ablation strategies were less-well-defined in these studies.

### 4.5. Predictors of AF/AT Recurrence

Whereas earlier studies found CHA2DS2VASc-Score, existence of LVZs, and LA size as predictors of recurrence after a redo procedure [15,16,17,18], age was the only significant predictor in our study.

This result is in line with a study on first VGA procedures out of our group, where age ≥ 68 was also an independent predictor of recurrences [8]. Behind the predictive value of a higher age might be the increased potential to faster disease progression during further follow-up [10].

Furthermore, there was a trend to more applied lines during the initial procedure in patients with recurrence. Knowing that lines were only applied in cases of LVZs, the increased amount of fibrosis might be the causal factor. The number of patients with LVZs was only numerically higher in this cohort, but the result was not significant. However, quantitative analysis of LVZs was not performed.

The introduction of CLOSE-like ablation and HPSD 50W ablation did not influence recurrence rate. This is in line with the results of larger studies with AI-guided high-power short-duration versus low-power long-duration ablation [19,20].

Due to the small number of patients, this analysis is only exploratory and allows for no clear conclusions.

### 4.6. Limitations

The validity of the current study is limited by the small number of patients and the non-randomized setting. However, there are no randomized studies on VGA including redo procedure outcomes, nor are there any observational data for a purely persistent AF population.

Discontinuous ECG monitoring in most of the patients is a limitation of follow-up analysis in our study. Nevertheless, given that all patients had recurrent, persistent AF before ablation, then 72 h Holter monitoring with symptom-driven event recording is a valuable monitoring method.

### 4.7. Conclusions

Our study found that reconnection of lines and new or progressive LVZs are possible driving causes for recurrence after VGA in persistent AF cases in older patients. Increasing the durability of LA linear lines is an important future goal. However, redo ablations after VGA have good efficacy and can be performed safely.

## Figures and Tables

**Table 1:** Comparison of baseline parameters of total initial procedure cohort of first procedures with first redo procedure cohort.

Parameters	Total Cohort of Initial Procedures *N* (%), M ± SD *N* = 683	Total Cohort of First Redo Procedures *N* (%), M ± SD *N* = 77	*p*
Age (years)	68.4 ± 8.9	70.2 ± 8.2	0.069
Sex (male)	381 (55)	39 (48)	0.391
Diabetes mellitus	193 (28)	21 (26)	0.819
Hypertension	571 (83)	64 (79)	0.891
Persistent	683 (100)	77 (100)	1
BMI (kg/m^2^)	29.3 ± 5.5	29.5 ± 4.8	0.661
GFR (mL/min)	66.2 ± 19.3	65.5 ± 17.5	0.745
LVEF (%)	52.5 ± 11.1	54.2 ± 9.6	0.136
LAESVI (mL/m^2^)	41.2 ± 12.8	42.7 ± 12.1	0.408
CHA2DS2Vasc (Points)	3.3 ± 1.5	3.4 ± 1.4	0.584

GFR: glomerular filtration rate. LVEF: left ventricular ejection fraction. LAESVI: left atrial end-systolic volume index.

**Table 2:** Necessary ablation approaches for initial procedure and first redo procedure.

Procedural Data	Total Cohort (*n* = 77) *n* (%)
PVI-only initial ablation procedure	49 (64)
PVI plus LVZ ablation initial procedure	28 (36)
Re-PVI-only 1st redo	21 (27)
LVZ-only ablation 1st redo	35 (45)
Re-PVI plus LVZ ablation 1st redo	17 (22)
PVI-only initial ablation and 1st redo	24 (31)
New LVZs without reconnections of PVs/lines	13 (16.9)

LVZs: low-voltage zones. PVI: pulmonary vein isolation. Re: redo.

**Table 3:** Comparison between the patients with and without recurrence off drugs.

Parameters	Recurrence of AF or AT *N* (%), M ± SD *N* = 23	Free of Recurrence of AF or AT off Drugs *N* (%), M ± SD *N* = 46	*p*
Age (years)	73.3 ± 5.9	69.1 ± 8.9	0.049
Sex (male)	9 (39)	24 (52)	0.444
BMI (kg/m^2^)	28.4 ± 4.3	30.3 ± 5.0	0.141
Cardiomyopathy *	10 (44)	12 (26)	0.176
Diabetes mellitus	7 (30)	10 (22)	0.555
Hypertension	18 (78)	40 (87)	0.487
LVEF (%)	54 ± 9	55.6 ± 8.8	0.400
LAESVI (mL/m^2^)	46.1 ± 10.8	40.7 ± 11.6	0.114
CHA2DS2Vasc (Points)	3.7 ± 1.4	3.2 ± 1.5	0.181
LVZs initial procedure	13 (57)	18 (39)	0.205
LVZs ablation initial procedure	11 (48)	13 (28)	0.119
Lines per patient during initial procedure	1.0 ± 1.3	0.5 ± 1.0	0.082
New LVZs 1st redo procedure	7 (30)	16 (35)	0.791
Progressive LVZs 1st redo procedure	4 (17)	10 (22)	0.760
New or progressive LVZs 1st redo procedure	11 (48)	26 (57)	0.610
LVZs 1st redo (%)	19 (83)	31 (67)	0.256
Reconnected PV % of PVs in 1st redo	70 (19)	38 (22)	0.519
Reconnected lines % of applied lines 1st redo	8 (35)	12 (50)	0.657

BMI: body mass index. LVEF: left ventricular ejection fraction. LAESVI: left atrial end-systolic volume index. LVZs: low-voltage zones. PVs: pulmonary veins. * Definition of Cardiomyopathy: EF < 55% or hypertrophic cardiomyopathy.

**Table 4:** Baseline parameters of patients undergoing a second redo procedure.

Parameters	Second Redo Cohort (*n* = 11) *n* (%), M ± SD
Age (years)	75 ± 5
Female (%)	8 (73)
BMI (kg/m^2^)	29.1 ± 4.9
Short persistent (%)	11 (100)
Cardiomyopathy * (%)	1 (9)
Arterial hypertension (%)	10 (91)
GFR (mL/min/1.7)	66.5 ± 13.5
Diabetes (%)	3 (27)
LVEF (%)	56.7 ± 5.7
LA-area (cm^2^)	26.7 ± 6.8
LAESVI (mL/m^2^)	48.3 ± 16.5
CHA2DS2Vasc (points)	3.9 ± 1.0

GFR: glomerular filtration rate. LVEF: left ventricular ejection fraction. LA-area: left atrial area. LAESVI: left atrial end-systolic volume index. * Definition of Cardiomyopathy: EF < 55% or hypertrophic cardiomyopathy.

**Table 5:** Procedural findings and ablation approaches of second redo procedures.

Procedural Data	Second Redo Cohort (*n* = 11) *n* (%), M ± SD, Median (Min; Max)
Reconnected PVs per Patient	0.3 ± 0.7
Reconnected PVs of all PVs	3/44 (7)
Reconnected lines per patient	0.7 ± 0.6
Reconnected lines of all lines	8/19 (42)
New LVZs	1 (9)
Progressive LVZs	5 (45)
PVI	1 (9)
LVZ ablation	8 (73)
PVI + LVZ ablation	1 (9)
Other ablation techniques	1 (9) *

* 2xCTI, 1xSVC, 1xAVNRT ablation additional. AVNRT: atrioventricular nodal reentry tachycardia. CTI: cavotricuspid isthmus. PVs: pulmonary veins. LVZs: low-voltage zones. PVI: pulmonary vein isolation. SVC: superior vena cava.

## Data Availability

The data shared is in accordance with consent provided by participants on the use of confidential data. The publication of the data of this manuscript does not compromise the anonymity of the participants or breach local data protection laws.
